# Comprehensive repeatome annotation reveals strong potential impact of repetitive elements on tomato ripening

**DOI:** 10.1186/s12864-016-2980-z

**Published:** 2016-08-12

**Authors:** Ophélie Jouffroy, Surya Saha, Lukas Mueller, Hadi Quesneville, Florian Maumus

**Affiliations:** 1 URGI, INRA, Université Paris-Saclay, 78026 Versailles, France; 2 Boyce Thompson Institute, Ithaca, NY 14853 USA; 3 Department of Plant Breeding, Cornell University, Ithaca, NY 14853 USA

**Keywords:** Fruit ripening, DNA methylation, Transposable elements, Tomato

## Abstract

**Background:**

Plant genomes are populated by different types of repetitive elements including transposable elements (TEs) and simple sequence repeats (SSRs) that can have a strong impact on genome size and dynamic as well as on the regulation of gene transcription. At least two-thirds of the tomato genome is composed of repeats. While their bulk impact on genome organization has been recently revealed by whole genome assembly, their influence on tomato biology and phenotype remains largely unaddressed. More specifically, the effects and roles of DNA repeats on the maturation of fleshy fruits, which is a complex process of key agro-economic interest, still needs to be investigated comprehensively and tomato is arguably an excellent model for such study.

**Results:**

We have performed a comprehensive annotation of the tomato repeatome to explore its potential impact on tomato genome composition and gene transcription. Our results show that the tomato genome can be fractioned into three compartments with different gene and repeat density, each compartment presenting contrasting repeat and gene composition, repeat-gene associations and different gene transcriptional levels. In the context of fruit ripening, we found that repeats are present in the majority of differentially methylated regions (DMRs) and thousands of repeat-associated DMRs are found in gene proximity including hundreds that are differentially regulated. Furthermore, we found that repeats are also present in the proximity of binding sites of the key ripening protein RIN. We also observed that some repeat families are present at unexpected high frequency in the proximity of genes that are differentially expressed during tomato ripening.

**Conclusion:**

Altogether, our study emphasizes the fractionation as defined by repeat content in the tomato genome and enables to further characterize the specificities of each genomic compartment. Additionally, our results present strong associations between differentially regulated genes, differentially methylated regions and repeats, suggesting a potential adaptive function of repeats in tomato ripening. Our work therefore provides significant perspectives for the understanding of the impact of repeats on the maturation of fleshy fruits.

**Electronic supplementary material:**

The online version of this article (doi:10.1186/s12864-016-2980-z) contains supplementary material, which is available to authorized users.

## Background

The majority of plant genomes contain a large fraction of repetitive DNA, collectively referred to as the repeatome of a species [1]. The major types of repetitive elements in plant genomes comprise transposable elements (TEs), simple sequence repeats (SSRs), and ribosomal DNA. The de novo detection of repeated sequences also commonly reveals the significant contribution of repeated features that remain unclassified. Because of their relatively high duplication rate, TEs, which mediate their own transposition, are generally the most abundant sequences in plant repeatomes. While TE insertions can be deleterious by disrupting genes, mounting evidences demonstrate that some TE copies can also impact the transcriptional regulation of nearby genes and can thereby generate adaptive traits and phenotypes of agro-economical interest [2].

TEs can impact the transcription profile of proximate genes by a variety of means, at the structural and quantitative levels. For instance, TE sequences can distribute new regulatory regions such as promoters [2]. Being repetitive across genomes, TEs can also build regulatory networks that influence the expression of several genes in a coordinated fashion [3]. TEs can also provide alternative transcription start sites and other transcript isoforms [4]. Furthermore, TE sequences are commonly modified by the cells by the addition of methyl groups on cytosine residues in a process called DNA methylation, that causes local genome compaction and prevent TE transcription [5]. This epigenetic regulation can occasionally act in cis or spread into neighboring genes and affect their expression [6, 7].

Nevertheless, the potential impact of TEs and other repetitive elements remain to be addressed in a comprehensive manner in most plant species at different developmental stages and in a variety of tissues. Of marked interest in agronomy, the role of repeated sequences in the ripening of fleshy fruits remains to be investigated in a comprehensive manner. Tomato, S. lycopersicum, is the most cultivated fleshy fruit/vegetable worldwide with a global production around 160 million tonnes each year (http://faostat3.fao.org). The genome of the inbred tomato cultivar ‘Heinz 1706’ was sequenced and assembled in 2012, and TEs were found to make a large contribution to the nearly complete assembly of this ~900 megabases (Mb) genome. Previous reports have demonstrated that TEs do play roles in the determination of fruit morphology and quality [8, 9]. In addition, a recent study has investigated the changes of the tomato methylome at single-base resolution and has identified thousands of regions that present dynamic methylation patterns, mostly hypomethylation, during fruit ripening [10]. These differentially methylated regions (DMRs) were found to associate with differentially expressed genes in maturing tomatoes and with binding sites of the RIN (ripening inhibitor) MADS-box transcription factor which is a key regulator of ripening [11].

Here, we have investigated the global impact of repetitive elements on the tomato genome and their potential role in the orchestration of tomato ripening. By studying the composition of the genome in terms of genes and transposable elements contents, we show that it could be divided into three types of regions, each showing specific properties in genes and repeat content. We also found that globally the presence of repeated sequences near genes could slightly influence their expression, but that their methylation could instead have an impact that is still poorly defined. Finally, a comparison of the different stages of maturation reveals that the expression of genes in this process may be partly regulated by TEs and differentially methylated regions (DMRs).

## Results

### Comprehensive annotation of the Heinz 1706 repeatome

**Fig. 1:**
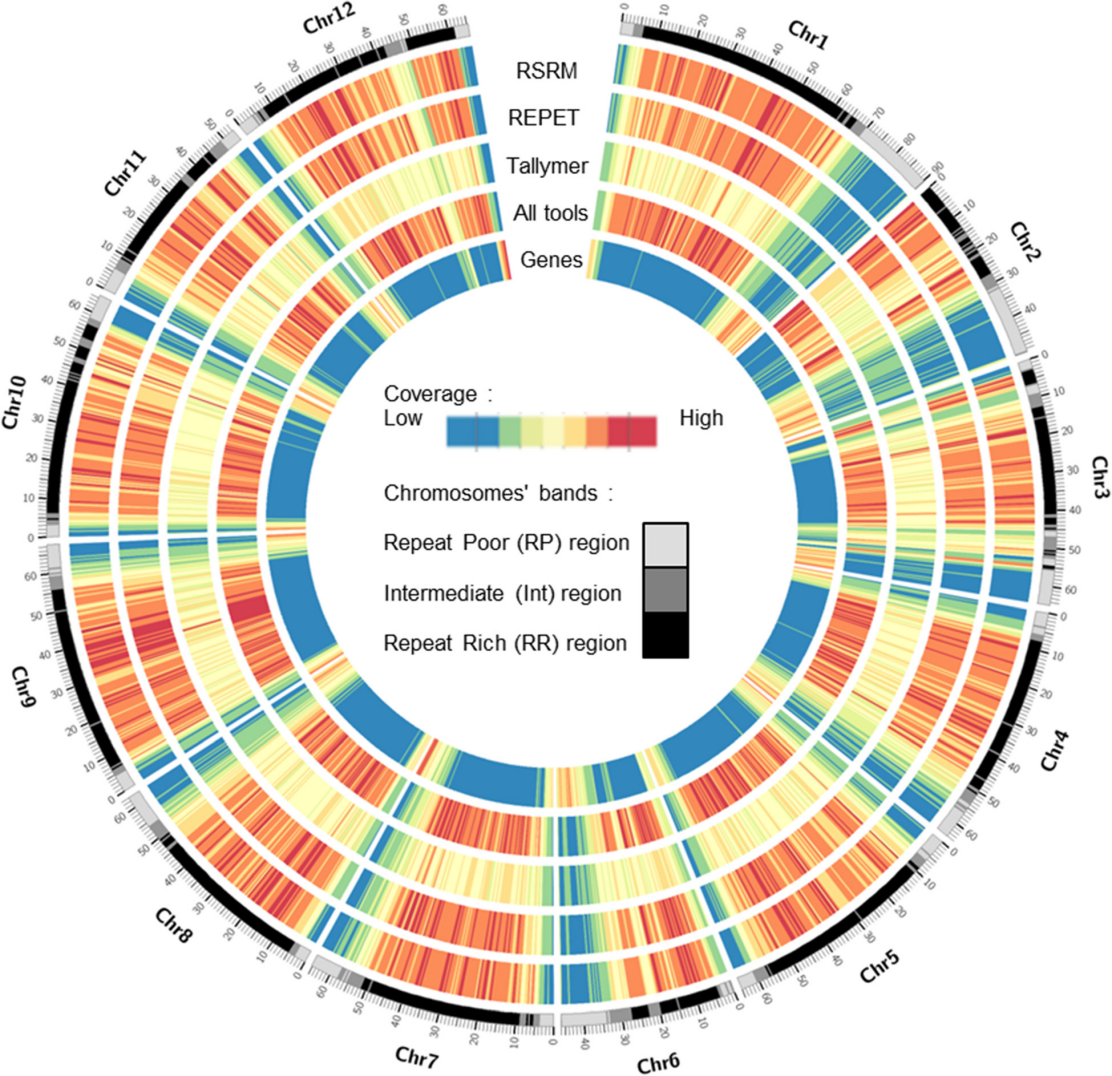
The tomato repeatome. Repeats and genes coverage by 500 kb windows with an overlap of 50 kb. Repeats annotation was conducted with three different tools: RepeatScout+RepeatMasker (RSRM), REPET, and the k-mer strict program Tallymer. Three types of genomic regions are visible in the chromosome bands and chromosome name and scale (in Mb) are indicated on the outer rim

The initial annotation of repetitive elements in the tomato genome has relied on the identification of representative sequences based on the presence of structural features [10, 12] and more recently on the use of a de novo repeat identification tool [13]. We have recently shown the advantage of combining different approaches in order to improve repeatome annotation in genomes [1]. Here, in order to generate a comprehensive annotation of the tomato repeatome, we have employed a combination of similarity- and k-mer-based methods with the REPET and RepeatScout programs, respectively for the de novo construction of libraries of consensus sequences representative of repetitive elements. Alignment-based annotation of the tomato assembly using these libraries yields 68 % (532 Mb) coverage and 72 % coverage of the non-gapped assembly (Fig. 1). For comparison, this annotation covers 96 % of the initial repeat annotation (reciprocally 82 %) and 95 % of the specific annotation of MITE elements established previously [10, 12]. It also covers 96 % of a recently published de novo repeat annotation [13] (reciprocally 84 %). In addition, we have employed a strict mapping of frequent k-mers in the genome assembly in order to detect short repeats that would have been missed by alignment-based strategies. This approach identified 292 Mb of perfect repeats, including 22 Mb that were not detected above. In total, our combined annotation covers 75 % of the ungapped tomato genome assembly.

In line with previous findings [12], we found that LTR-retrotransposons are the most abundant TEs in the Heinz 1706 assembly with Gypsy- and Copia-type elements representing 45 and 14 % of the repeat annotation, respectively (Additional file 1: Figure S1, Additional file 2: Table S1). In addition, Class 2 elements (also called DNA transposons), including both autonomous and non-autonomous elements, were found to contribute 5 % of the repeat annotation. Furthermore, environmental viruses, which can happen to integrate into plant DNA and being vertically transmitted over generations in the form of endogenous viral elements (EVEs), can also represent significant constituents of their nuclear genomes [14, 15]. In tomato, we found that EVEs, including members of the recently described Florendovirus [15] and Mitovirus [16], contribute over 4 Mb of the Heinz 1706 assembly. Finally, SSRs and unclassified repeats were observed to make a substantial contribution to the repeatome annotation (Additional file 1: Figure S1, Additional file 2: Table S1). All the annotations generated here are available at the Sol Genomics Network.

### Determining three genomic compartments with contrasted repeatome composition

Plotting the density of repeats and genes along the chromosomes confirms that distal chromosome regions are gene-rich while the remainder of the genome is densely populated by repetitive elements (Fig. 1). Clustering of the repeat and CDS densities in 500 kilobases (kb) windows allowed empirically determining three kinds of genomic regions (Additional file 3: Figure S2; Additional file 2: Table S2): repeat-rich (485 Mb), repeat-poor (161 Mb), and a third, intermediate category (113 Mb), hereafter referred to as RR, RP and INT compartments, respectively. In line with the repeatome distribution along the chromosomes, INT regions are most of the time positioned in transition zones between RR regions, which are found in pericentromeres, and RP regions, located at the chromosome tips.

**Fig. 2:**
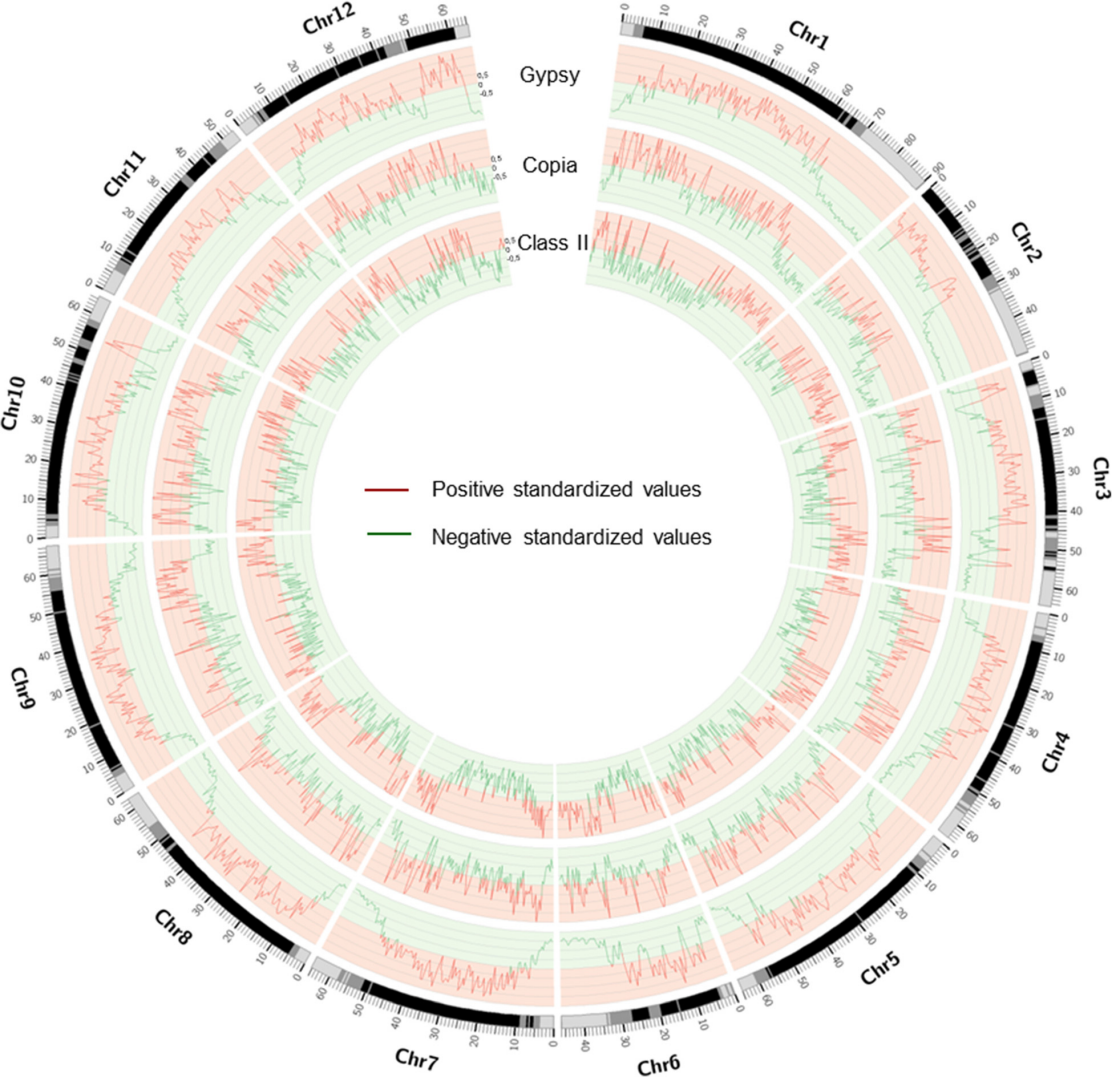
Genome coverage for different repeats families. This graphic represents the standardized values of coverage of the three major TE families in tomato genome, Gyspsy, Copia and Class II elements. The coverage is calculated by window of 500 kb with an overlap of 50 kb and standardized values are determined based on these calculations. Positive values (in red) reflect an enrichment while negative values (in green) reflect depletion. Chromosome name and scale (in Mb) are indicated on the outer rim. The interval between two lines on the graph corresponds to a value of 0.5

The high abundance of repetitive elements, and especially TEs, in the Heinz 1706 genome to a large extent determines the composition of intergenic DNA. We therefore addressed whether the distribution of different types of TEs is homogeneous along the tomato chromosomes. Remarkably, applying a local enrichment statistical analysis [17], we observed a differential distribution of the main types of TEs between the three compartments established above (Fig. 2; Additional file 4: Figure S3). For instance, Copia-type LTR retrotransposons (LTR-RT) are enriched in INT regions and depleted in RP regions. In contrast, Gypsy-type LTR-RTs are predominant in the RR space and under-represented in the RP and INT regions. In addition, Class II elements (autonomous and non-autonomous DNA transposons) are enriched in the RP and INT regions and depleted in the RR compartment. This contrasted distribution of the tomato repeatome in the three genomic compartments may have significant impact on DNA composition. In fact, we observed that the G + C content in the repeatome (REPET + RepeatScout) of each compartment is also different by being relatively higher in RR than in RP, INT showing an intermediate value (Additional file 5: Figure S4A). Correlatively, we found that among the main TEs in tomato, Gypsy-type elements have the highest G + C content followed by Copia-type elements then DNA transposons (Additional file 5: Figure S4B). A similar bias in repeatome composition has been described in *Arabidopsis thaliana* [18] although the causes seem to be different in the two genomes.

### Contrasted gene density between genomic compartments

As predictable, we found that gene density is much lower in RR and INT regions (15 and 57 genes per Mb, respectively) as compared to RP regions (122 genes per Mb). Nevertheless, RR and INT regions contain an important number of predicted genes (7554 and 6466, respectively, i.e. approx. 40 % of the total gene count). Yet the majority of the predicted genes (19,781) are located in RP regions.

Protein-coding TEs are commonly confounded by gene prediction programs, leading to gene models that actually correspond to TEs. We therefore took advantage of this new genome annotation to address the potential contribution of TE-genes to the set of predicted genes in each genomic compartment. We first established a set of confidently classified TEs comprising repetitive elements with similarity to known TEs and/or with TE domains and no other conflicting evidence using the TE classifier PASTEC [19]. The comparison of their positions with those of predicted CDS allowed the identification of 2246 putative TE-genes for which over half of the CDS fraction is covered by high confidence TEs (Additional file 2: Table S3). While most TE-genes are located in RR and INT compartments, these areas still contain a high number of non-TE genes that are distributed in a highly repeated environment in contrast to those that are positioned within the RP space (Additional file 6: Figure S5A). Comparing the expression in leaves using available data [10] of TE-genes and non-TE-genes in different genomic regions showed a significant difference between these two groups. Indeed, while the expression of TE-genes remains at a zero mean, or practically, that of non-TE-genes is contained in a large range of values regardless of the genomic compartment (Additional file 6: Figure S5B). This observation suggests possible TE contamination in the predicted gene set so we decided to exclude putative TE-genes of further analyzes.

### Correlation between repeat density and gene expression

The genes that are located in the genomic compartments that we defined on the basis of repeat density appear to be positioned within strikingly different environments in terms of genome dynamics and composition at the chromosome scale. We further examined whether these contrasted landscapes correlate with distinct properties of the gene sets from each compartment including distance to repeats, evolutionary origin, and expression levels.

The different TE densities in the three genomic compartments suggest the respective gene sets may be in different proximity to repeats. For each compartment, we measured the distance from genes to the closest upstream or downstream genomic repeats. As expected, we found that on average, genes from the repeat-rich and intermediate compartments have closer upstream and downstream repeats as compared to those located in the repeat-poor regions (medians: RR = 420, INT = 442, RP = 640; Mann-Withney U test (MWU) [RR vs RP] *P* value < 0.0001; MWU [INT vs RP] *P* value < 0.0001, for upstream and downstream repeats) (Additional file 7: Figure S6). Surprisingly, gene sets from the repeat-rich and intermediate compartments show rather similar distances to proximal repeats. More interestingly, we also observe for each compartment that upstream repeats are closer to genes than downstream repeats, especially in the RP compartment.

**Fig. 3:**
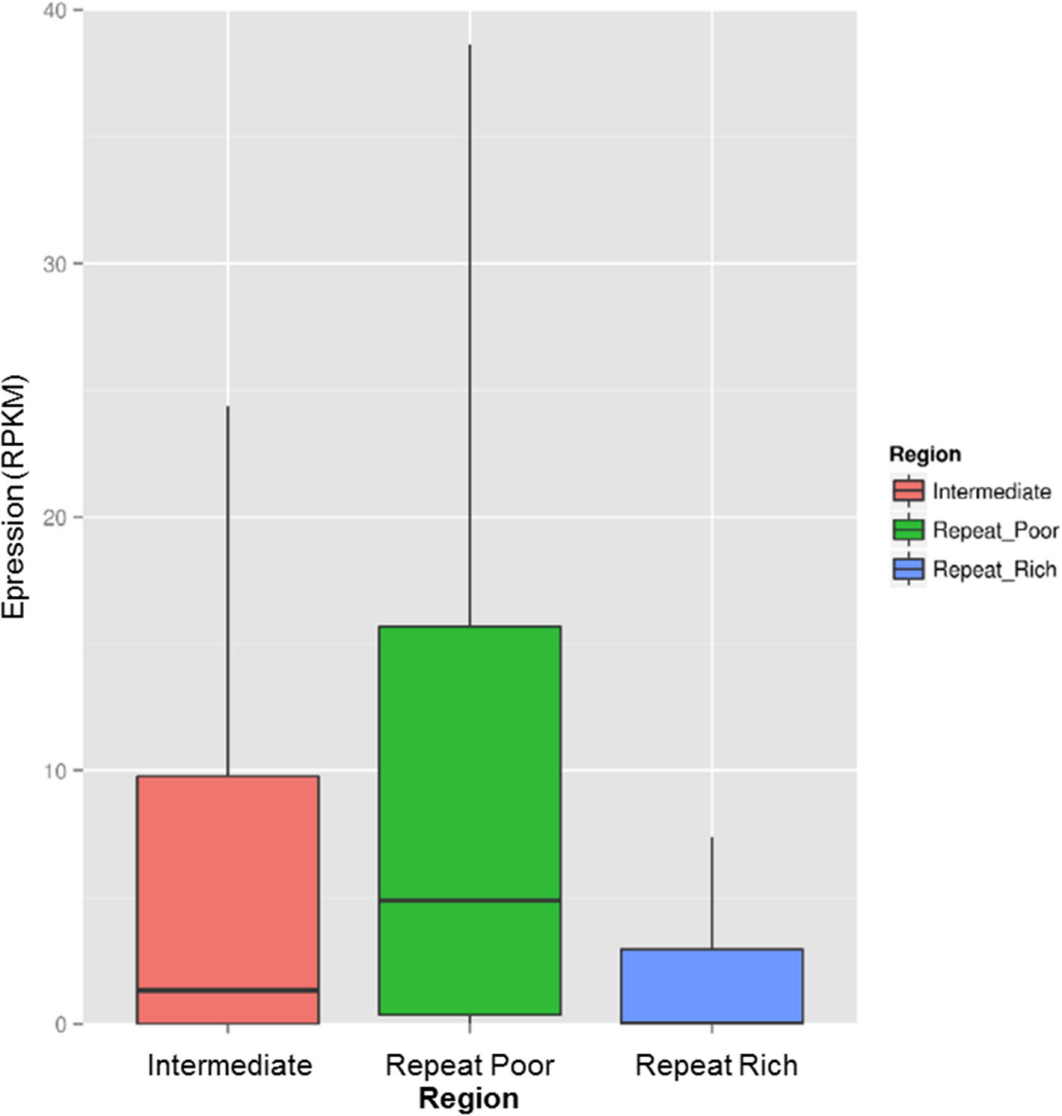
Global expression in leaf in the three genomic regions. In RR region, median value is equal to 0 and only 1567 (out of 7529) genes have a RPKM value higher than five, while in RP region, median value is equal to 4,87 and 9796 (out of 19780) have a RPKM value higher than five, and in INT region median value is equal to 1,34 with 2299 (out of 6486) genes with a RPKM value higher than five

Because proximal genomic repeats can impact gene expression by a variety of means, we next addressed whether overall, genes within the different genomic compartments may present distinguished expression levels in tomato leaves. We found that, overall, genes from the RR, INT and RP compartments show strikingly different expression levels. Remarkably, the predicted genes from the RR set show very low median RPKM value and relatively few highly expressed genes (median-RR = 0,00; 1567 (21 %) with RPKM > =5) as compared to those located in the INT and RP regions (Fig. 3). Overall, we observe a strong negative correlation between repeat-density and the transcriptional levels of predicted genes (Mann Witney U test *P* value < 0.001 between each compartment). The observation that genes with closer repeat proximity show lower expression levels could reflect different biological and evolutionary phenomena. For instance, gene expression levels may be impacted by the presence of neighboring, especially upstream repeat-associated heterochromatin that may hamper the access of factors that initiate transcription. Another non-exclusive explanation could be that the different genome compartments enclose gene sets which expression is differentially regulated overall; for instance if constitutively and/or highly expressed genes are over-represented in repeat-poor regions or if stress-responsive genes are mostly present in RR.

### Correlation between repeat proximity and gene expression

**Fig. 4:**
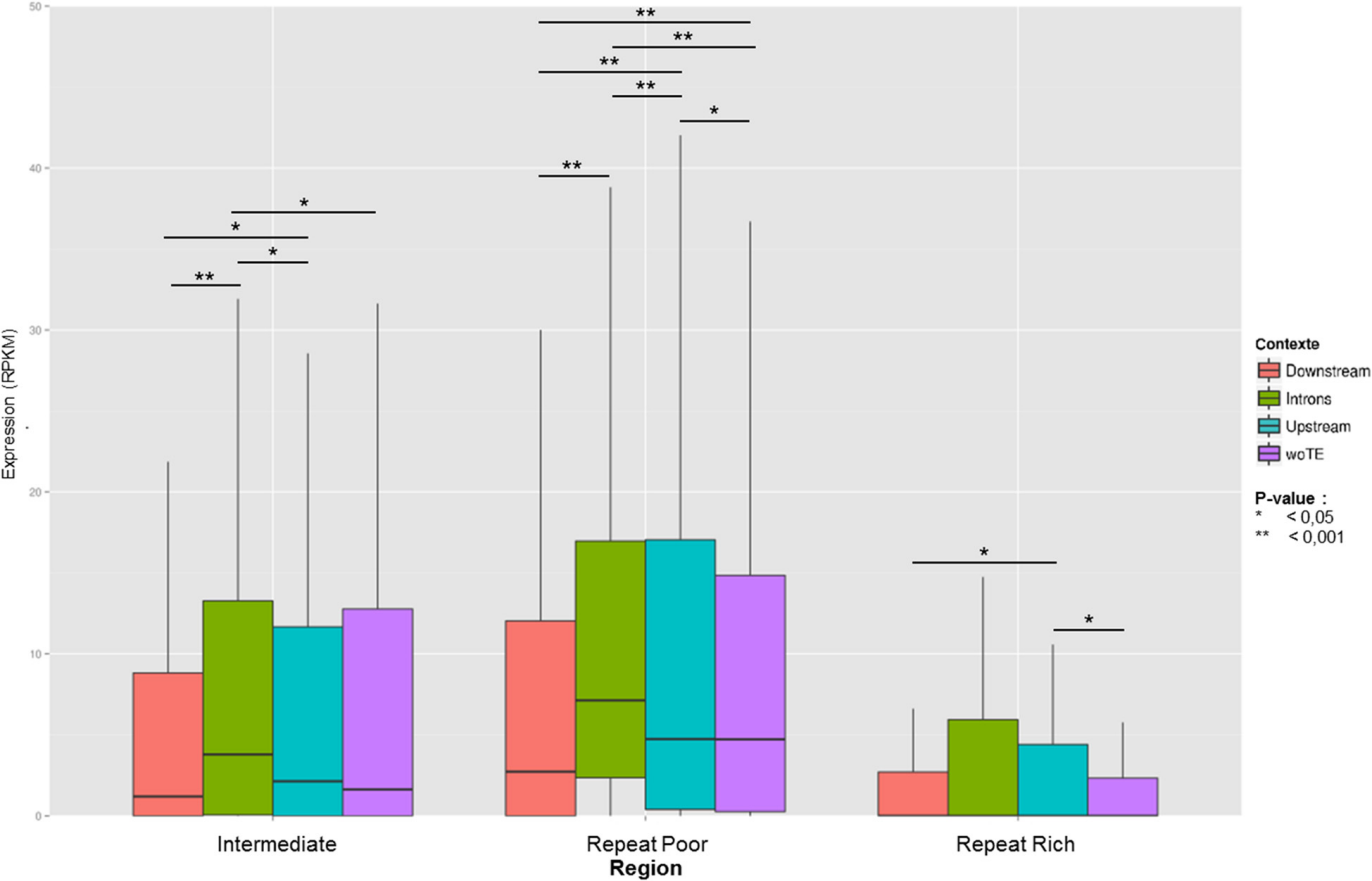
Gene expression in leaf considering repeat location and genomic region. Considering four different types of associations between genes and repeats, we observe the difference of expression between these categories. Statistical analyzes (Mann – Whitney U tests) were performed to validate the observations and these results are represented by the *P*-value on the graphic

To investigate the overall differences in gene expression levels observed between genomic compartments, we first addressed whether repeat proximity has a predominant effect on gene transcription levels. Indeed, assuming that repeated elements are generally methylated in the genome [10] and are thus predominantly associated to regions of condensed chromatin, one could expect a negative impact of repeats on the transcription of nearby genes and hence a negative correlation between repeat distance and gene expression levels. However, we could not detect such an overall negative correlation when comparing the expression levels of genes with upstream repetitive element in different 100 bp bins of a 1 kb window (data not shown). We then reason that such a signal could be biased by other gene-proximal repeated elements that may also affect gene expression. Indeed, repeated elements may be positioned upstream, downstream and within introns of the same gene, and all these configurations may influence gene expression by a variety of means [2]. We therefore examined gene expression levels following the presence of repeated elements exclusively in one of the above mentioned configuration. In leaves, we found that the genes without upstream repeats in 1 kb present lower transcript levels than those with an upstream repeat in RR and RP (MWU of RPKM values [No repeat vs 1 kb upstream], in leaves: *P* value = 0.005 in RR, *P* value = 0.279 in INT and *P* value = 0.003 in RP) (Fig. 4). Also, the genes with intronic repeats show robust statistical support of highest median transcript values in the RP and INT compartment (MWU of RPKM [Intronic vs No repeat] in leaves: *P* value = 0.088 in RR, *P* value = 0.008 in INT and *P* value < 0.001 in RP).

To gain more insights on the overall local effects of repeats on gene expression, we specifically analyzed the impact of the DNA methylation of repeats, which can alter the expression of nearby genes. Interestingly, we found that, in leaves, repeat methylation is associated with decreased gene transcript levels when located in an intron or upstream of the gene in RR and INT (MWU of RPKM [methylated vs non methylated repeat]: 0.05 > *P* value > 0.001) (Additional file 8: Figure S7). Similarly, in INT and RP, an association between gene expression levels and the presence of methylated repeats is visible if they are located downstream, upstream or in an intron of the gene (MWU of RPKM [methylated vs non methylated repeat]: 0.05 > *P* value > 0.001). However, we observed that methylated or unmethylated proximal repeats can be associated with globally higher or lower gene expression levels during the different stages of maturation in different genomic regions (data not shown).

### Different gene categories between genomic compartments

**Fig. 5:**
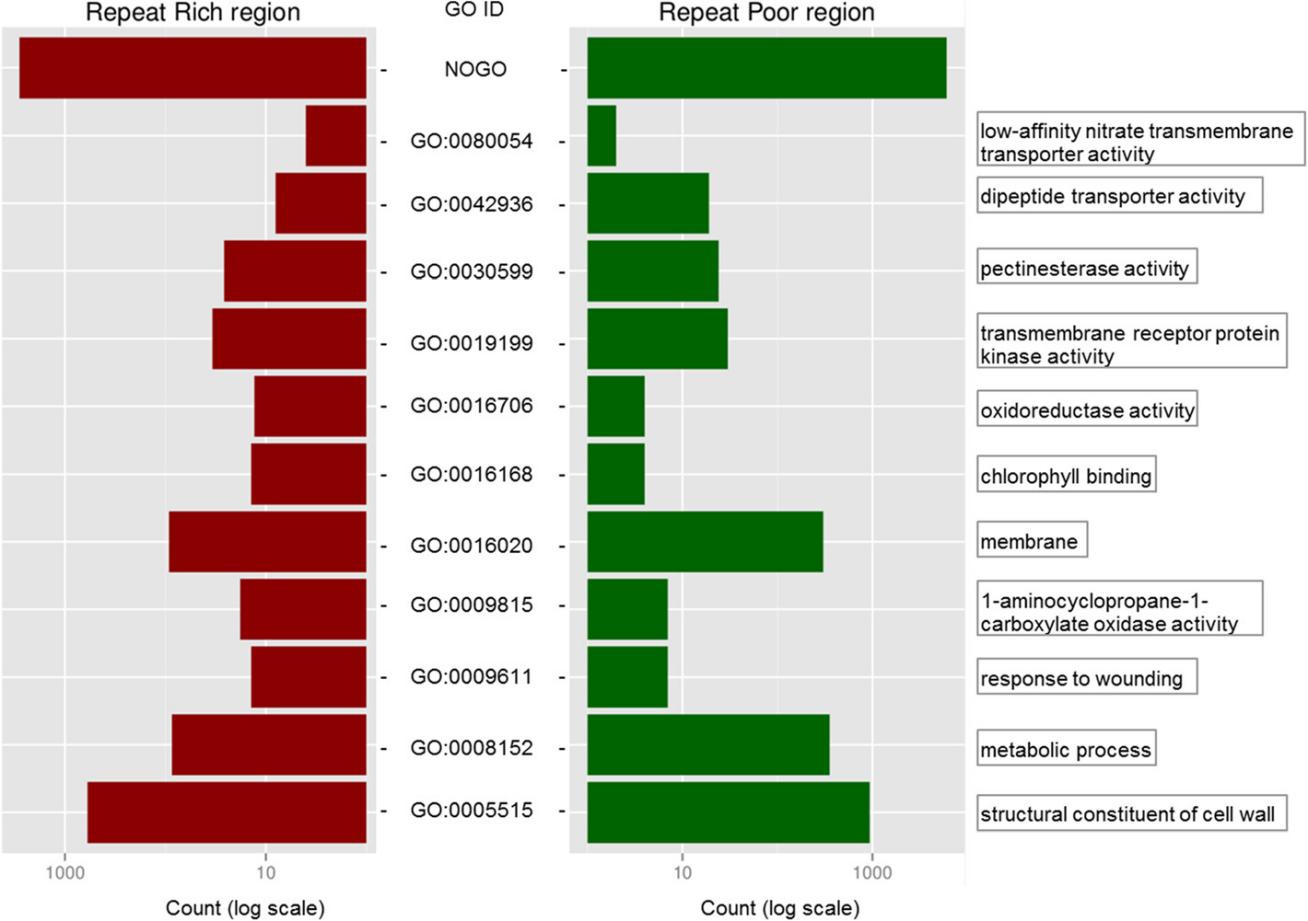
GO terms distribution between genomic regions. Counting of each GO term in the RR, INT and RP regions give us the possibility to compare their composition in terms of predicted gene functions

We also addressed whether the different genomic compartments may contain genes with a biased composition of predicted function. For example, we found that the gene ontologies GO:0005515 (protein binding), GO:0047714 (galactolipase activity) and GO:0009611 (response to wounding) are differentially represented in the RP-vs-RR, RP-vs-INT and INT-vs-RR compartments, respectively (chi-square *P* value < 0.001 with Bonferroni correction) (Fig. 5; Table 1). In addition, genes without functional category are more frequent in RP regions than in RR and INT. A number of stress genes have been characterized and predicted in tomato (see [20]), which expression profiles are typically expected to be stress-induced. We found that most of these genes (2056) are in RP, while INT contains 604 of these genes and RR only 371. While this distribution corresponds to the expectation if randomly distributed in RP and INT, the number of stress genes in RR is lower than expected (chi-square *P* value = 0.84321 (degree of freedom X-square = 0.04) in RP vs others, *P* value = 0.193 (X-square = 1.70) in INT vs others and *P* value < 0.001 (X-square = 168.25) in RR vs others).

**Table 1:** GO terms repartition in the three compartments of the genome. Exemple of deviation of repartition of fifteen GO terms between the three genomic regions in the tomato genome. Column ‘*P*-value’ indicates results from the statistical analyzes (chi-square test) while column ‘counting’ gives the number of a GO term in a specific region

GO	*P*-value			Counting		
	RR vs INT	RR vs RP	INT vs RP	RR	INT	RP
GO:0005199	0	0	1	15	1	3
GO:0005515	0,076	0	0,003	596	644	935
GO:0008152	0,03	0	0	86	139	354
GO:0009611	0	0,003	0,099	14	0	7
GO:0009815	0	0	0,278	18	1	7
GO:0016020	0,002	0	0,385	92	165	303
GO:0016168	0,002	0	1	14	2	4
GO:0016706	0,042	0	0,43	13	5	4
GO:0019199	0	0,001	0,424	34	13	30
GO:0030599	0,572	0,008	0	26	37	24
GO:0042936	0,001	0,844	0	8	34	19
GO:0080054	0,003	0,192	0	4	23	2
NOGO	0,009	0,001	0,761	2834	3570	6037

We also investigated whether the genes positioned within the different compartments may show different evolutionary origins. In this scope we used three sets of genes that derive from phylogenetic reconstructions of the gene sets from dozens of plant genomes including three members of Asterids (*Mimulus guttatus*, *Solanum lycopersicum* and *Solanum tuberosum*) [21]. The first set comprises the genes that were present in the Asterid ancestor (ANC2), the second set includes the genes existing in the Solanum ancestor (ANC1), and the third set encompasses genes that are specific to tomato (NEW). Again, we observe significant differences between genomic compartments (Additional file 9: Figure S8). Interestingly, we found that the ANC2 gene set is enriched in the RP regions (chi-square *P* value < 0.001). Instead the Solanum-derived gene set and the tomato-specific genes are enriched in the RR regions (chi-square *P* value < 0.001 for both groups). Furthermore, the intermediate compartment is depleted for new genes and enriched in genes of Asterid origin (chi-square *P* value < 0.05 for both groups).

### Several repeat families are associated with differentially regulated genes during ripening

**Fig. 6:**
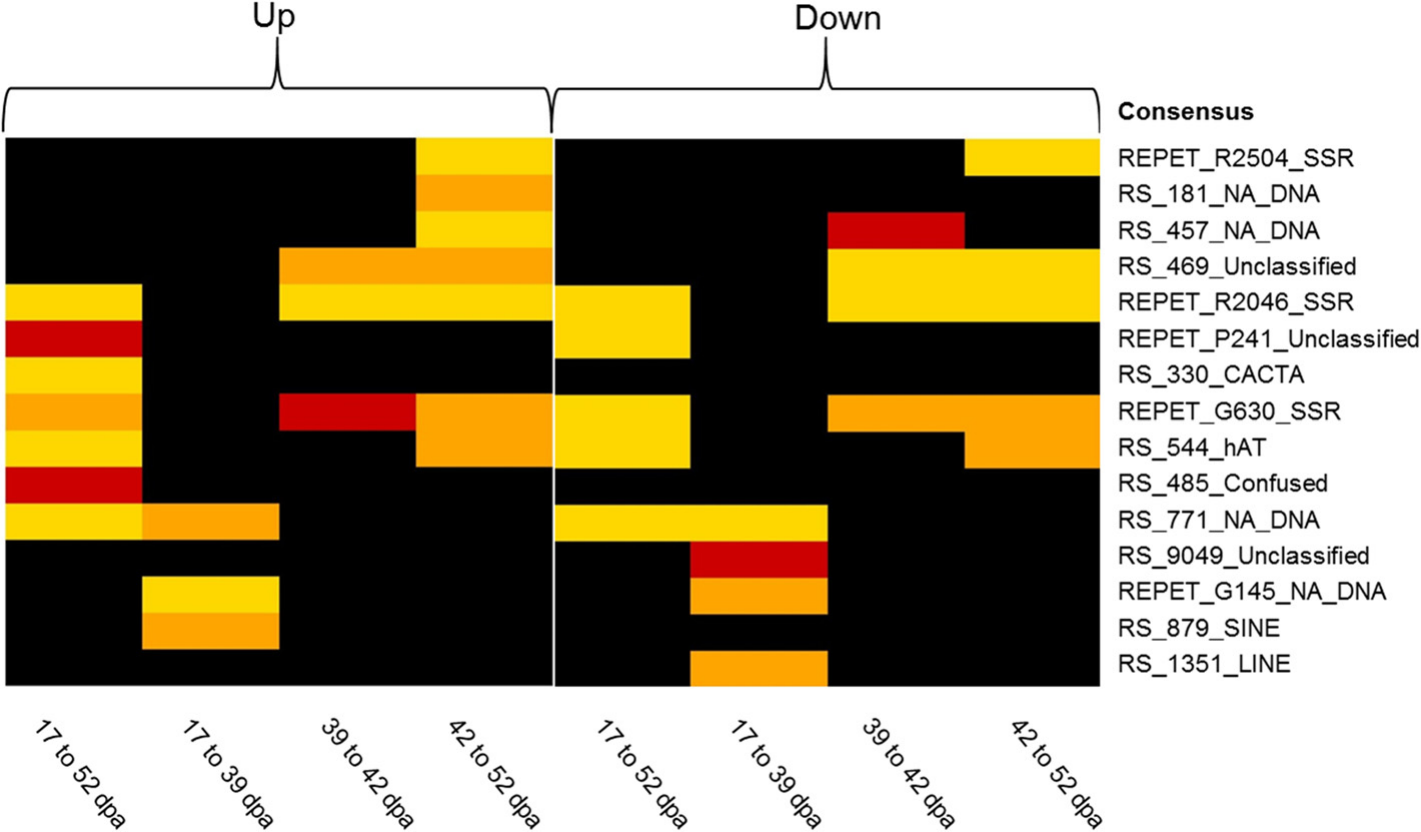
Enriched repeat families nearby differentially expressed genes. Fold enrichment of associations between consensus and differentially expressed genes during maturation. ‘Up’ table shows the fold enrichment of consensus within 1 kb of up-regulated genes, while ‘Down’ table represents the fold enrichment of consensus within 1 kb of down-regulated genes. A yellow box represents a 1-2.5 fold enrichment, orange box: 2.5-6 fold enrichment and red box: 6-9 fold enrichment. NA_DNA stands for « non-autonomous DNA elements »

Tomato fruit ripening is accompanied by successive changes in the regulation of thousands of genes as determined by comparison of transcriptomes from pericarps at four different stages of tomato maturation (17 d.p.a = Days Post Anthesis, 39 d.p.a, 42 d.p.a and 52 d.p.a) [10]. Because repeats can provide regulatory elements and can be involved in the establishment of gene regulation networks (see [3]), we have investigated whether copies of any of the tomato repeat consensus would be positioned nearby ripening-modulated genes more frequently than expected if repeat copies were distributed randomly among stable, up- and down-regulated genes (see [22]). Indeed, such distributions could reflect the specific retention and perhaps the selection and function of specific repeats in the proximity of the genes that are differentially regulated during ripening. Interestingly, we observed that 11 and 13 repeat families are present at high frequency compared to expectation nearby genes that are up and down regulated during ripening, respectively (Fig. 6, *P* values for chi-square with Bonferroni correction in Additional file 2: Table S4). These families comprise unclassified elements and SSRs as well as several putative class 2 (including autonomous and non-autonomous elements) and class 1 TEs (1 LINE and 1 SINE). Remarkably, several elements appear to be enriched both at up- and down-regulated genes, sometimes at different extent and timing during ripening.

### Repeats sustain the dynamic methylome during fruit ripening

Tomato ripening also goes together with thousands of changes in DNA methylation levels along the chromosomes that probably have a widespread impact on the regulation of gene expression [10]. Because repeats are generally methylated, we explored the potential contribution of repeat-associated methylation changes on gene transcriptional regulation through tomato ripening. In a dynamic perspective, we examined the presence of Differentially Methylated Regions (DMRs) and their potential link to TEs and genes using methylome data from four different stages of tomato maturation (17 d.p.a = Days Post Anthesis, 39 d.p.a, 42 d.p.a and 52 d.p.a) [10].

We first investigated the associations between DMRs and TEs. Among the 52,095 DMRs present in the Heinz 1706 genome, 72.29 % of them associate with genomic repeats (18.65 % of the DMRs overlapping repeats, 54.57 % are included in a repeat and 0.80 % have a repeat included in their sequence), which is a greater proportion than if DMRs were randomly distributed in the genome (chi-square *P* value < 0.001 and x-squared = 129.55). The genomic distribution of these associations shows that the majority (72.34 %) are in the RR region, while RP contains 14.23 % of the associations and INT only 13.43 %. Statistical analysis of this distribution shows that the observed values do not match those expected if repeat-associated DMRs were randomly distributed in the genome (chi-square *P* value < 0.001 in all three cases), RR being enriched in repeat-DMR associations while INT and RP are depleted. Looking at the repeats involved in the associations with DMRs, it appears that not all of them are involved in similar proportions (Additional file 10: Figure S9). Copia-type TEs are indeed more frequently associated with DMRs than expected (chi-square *P* value < 0.001), whereas DNA, non-autonomous DNA, Gypsy, and Line TEs as well as SSRs are observed in a number of associations lower than expected (chi-square *P* value < 0.001 for each family).

We have next explored the associations between repeat-associated DMRs and genes (upstream, downstream, and intronic). A total of 5021 associations could be found, the majority of them being in the RP region (54.39 %), the rest being distributed between INT and RR regions (22.19 % and 23.42 %, respectively). This distribution does not match the expected one (chi-square for *P* value < 0.001 for each comparison), the surrounding of RP and INT genes being enriched in repeat-associated DMRs in contrast with RR genes which are so depleted. Therefore, the expression of genes in medium and low repeat density regions is more likely to be influenced by repeat-associated DMRs than those located in RR. It was also observed that 42.12 % of the associations are made with an upstream repeat, 32.49 % have a downstream repeat and 25.39 % of the genes have a repeat-associated DMR in their intron. Among these associations, we found that some repeat families were unevenly represented. For instance, SSRs and Line-type elements are present in these associations more frequently than expected while Gypsy-type elements are less (chi-square *P* value < 0.001).

**Fig. 7:**
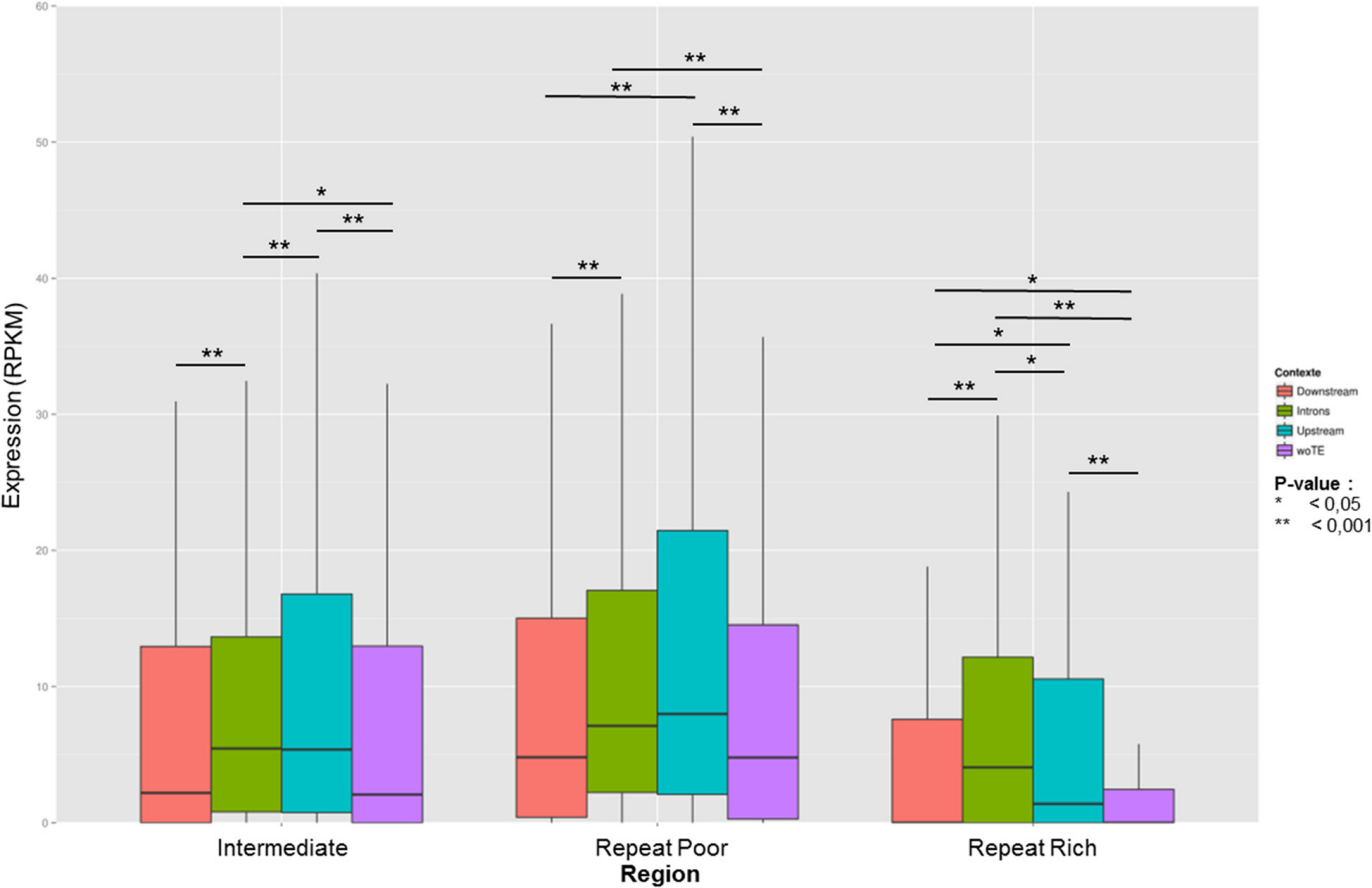
Expression in leaf of genes related to repeats-supported DMRs. Four types of associations between genes and repeats including a DMR were defined. A study of the expression of these genes was then carried out to assess the impact of the presence of methylated repeats on that expression. Mann–Whitney U tests were performed to validate the observations

Because of the high potential impact of repeat-associated DMRs on gene expression, we were interested in the genes in this configuration. By analyzing the expression of these genes in each region, we found significant differences (Fig. 7). Interestingly, we found that, in RR and RP regions, the expression of genes with repeat-associated DMRs upstream of their sequence or in one of their introns is higher than if the gene is isolated (MWU *P* value woTE vs Introns and woTE vs Upstream < 0.001). In INT regions, higher gene expression levels are observed only with upstream repeat-associated DMRs (MWU *P* value woTE vs Upstream < 0.001). In order to deepen this study, a more accurate analysis of associations involving differentially expressed genes during tomato ripening was performed. A total of 1773 differentially expressed genes associated with repeat-associated DMRs were found between stages 17 d.p.a and 52 d.p.a (754 upstream, 503 downstream, and 516 in introns). Comparing these observations to the proportions of associations from a random distribution of genes in the genome, we observed that the number of differentially expressed genes associated with repeat-associated DMRs is significantly lower than expected by chance (chi-square *P* value < 0.05). Investigating the contribution of different repeats, we noticed that DNA TEs and SSRs are more frequently associated with genes differentially expressed during ripening, while in contrast, Gypsy elements are less (chi-square *P* value < 0.001 with a Bonferroni correction).

RIN is a MADS-box transcription factor which directly regulates fruit ripening genes and RIN binding sites are typically adjacent to DMRs and hypomethylated during ripening, suggesting that neighboring methylation to a large extent determines RIN access to its binding sites [10]. We therefore explored more specifically the level of association between repeat-associated DMRs and the positions of RIN binding sites. First, we found that 67 RIN binding sites (1.63 %) overlap with DMRs, all of these DMRs being associated to repeats. DMRs are typically adjacent to RIN binding sites [10]. In total, we found that 512 RIN binding sites (12.4 %) associate with DMRs (including adjacent and overlapping), which is a greater fraction than observed in a random distribution of DMRs (chi-square *P* value < 0.001 and x-squared = 49.48). Furthermore, we found that 177 RIN binding sites (4.30 %) overlap or are adjacent to repeat-associated DMRs, which is a lower number than determined from a random distribution of DMRs (chi-square *P* value < 0.001 and x-squared = 215.98). Gypsy and SSR repeat families are involved in those associations in a different way than expected, Gypsy elements being less present (chi-square *P* value < 0.001 with a Bonferroni correction, 37 observed and 67 expected) and SSR elements being more present (chi-square *P* value < 0.001 with a Bonferroni correction, 31 observed and eight expected). Among these, 70 are associated with differentially expressed genes including 14 (Additional file 2: Table S5) of the 292 genes predicted as potential RIN targets [10].

## Discussion

The role of repeats in the regulation of gene expression [3] and many biological processes [2] has long been studied and is clearly demonstrated in some organisms. In this study we sought to understand their impact in the tomato genome and specifically the maturation process. Our various analyses, combining comprehensive repeat annotation, gene expression and DNA methylation data, help highlighting many aspects of the impact of repeats on tomato genome and biology.

Accordingly, we were able to demonstrate a compartmentalization of the genome as determined by its gene and repeat content. This separation into three major types of regions shows again the importance of repeats in shaping plant genomes. Indeed, through this division, we have shown that the content of each region is specific; both in terms of repeat content, some families are mainly located in one region or another, but also in terms of gene content, evolutionary origin and predicted function. Taken together, these observations may suggest a difference of dynamic of insertion and deletion, but also a differential control of transposable elements in the various ‘territories’ of the tomato genome. This aspect provides a new vision of the complexity of the tomato genome, especially in the case where a gene is found to be associated with different repeats. However, although the genes appear frequently associated to repeats, the presence of the latter doesn't have a clear effect on the levels of gene expression overall, and a negative correlation between the distance of repeats upstream and transcripts levels could not be established. Yet, it has been possible to observe variations of the expression taking into consideration the methylation status of the element. The *S. lycopersicum* genome has undergone limited repeat accumulation, the vast majority being ancient [20]. Therefore most ancestral repeat copies that have been retained in the tomato genome are likely to have almost neutral or adaptive effects while the most deleterious copies have likely been purified during evolution.

Thereafter, although a direct link between repeats and gene expression could not be determined, the analysis comparing different fruit maturation stages suggests a link between repeats and the presence of DMRs close to genes. This subset of DMRs could play a critical role in the regulation of gene expression, the repeats being the support of methylation and demethylation thereby influencing genome compaction at and nearby genes.. Most tomato ripening DMRs are linked to repeats and thousands of genes are adjacent to repeat-associated DMRs. Furthermore, 1773 genes that are differentially expressed during ripening are adjacent to repeat-associated DMRs, thereby suggesting a high potential impact of repeats (including both TEs and SSRs) on gene regulation and forecasting a certain degree of adaptiveness in gene-proximal repeat copies.

## Conclusion

Repeats seem to be a major element of the structure and organization of the tomato genome and are the significantly associated with methylation, some of these repeats being associated with activation of maturation genes in this organism [9]. Although the impact of specific TE copies on tomato biology is beyond the scope of this paper, further analysis by combining comparative genomics and transcriptomics will allow more targeted analysis of the contribution of repeats in tomato biology. Refining our results could also be considered by exploiting alternative transcripts and detection of other recurrent repeat-derived motifs near genes.

## Methods

### Repeat annotation

We used the TEdenovo pipeline [23] from the REPET package v2.2 with default parameters (with a minimum of five sequences per group) on the contigs of size > 100 kb in the SL2.40 assembly (representing approx. 340 Mb, gaps excluded) which generated a library of 818 consensus sequences. We used RepeatScout (version 1.0.5) [24] on the contigs of size < 100 kb with default parameters which generated a second library comprising 9085 consensus sequences. The whole SL2.40 assembly was annotated using the TEannot pipeline [25] from the REPET package v2.2 with the TEdenovo library as input. Blaster sensitivity was set to “3” and threshold scores were calculated for each consensus as the 99th percentile value of scores obtained against a simulated genome consisting of the reversed (not complemented) assembly (REPET annotation). The entire SL2.40 assembly was also annotated using RepeatMasker [26] with the RepeatScout library (RepeatScout annotation). Regions identified by the RepeatScout approach that span at least 50 bp and that are not included in REPET annotations (54 Mb) were combined to the latter. Tallymer was launched with a k-mer size = 16 and a minimum of 10 occurrences in the SL2.40 assembly. Fragments of at least 30 bp that do not overlap the REPET and RepeatScout annotations were kept. Consensus sequences from TEdenovo and RepeatScout were classified using the REPET dedicated utility released as the tool PASTEC [19]. Consensus classified as potential host genes because they contain host gene pfam domains (version 26.0) were excluded from this study. For the detection of TE genes, only the REPET annotations corresponding to TEs classified with high confidence (detection of non-conflicting evidences) were selected and compared to the CDS fraction of predicted genes. Genes which CDS are covered >50 % by selected TEs were categorized as putative TE-genes and not considered for further analysis.

### Repeat profile of the tomato genome

Genome coverage in genes (CDS) and transposable elements was calculated by 500 kb window with an overlap of 50 kb. Genes and transposable elements (REPET + RepeatScout + Tallymer) annotations and a karyotype were also needed.

The resulting files were formatted to be visualized graphically through the Circos software [27]. A clustering step of the different coverage windows by the k-mean method using R (*kmeans*), based on the gene content and repeat content in each window has allowed distinguishing three types of genomic regions. Finally, each TE and each gene was located on the genome. The repeats or genes that straddle two regions have been excluded.

### G + C content analysis

G + C content analysis was carried out at different scales in the genome: an analysis on the repeatome by genomic region and an analysis by repeat families in all regions. In each case, the sequence in FASTA format and the calculation of the G + C content was then performed by the command *infoseq*.

### GO term enrichment analysis

Each of the tomato gene annotation was associated with its GO term. These associations between genes and GO terms were then separated according to the genomic region to which they belong.

The expected values (noted EXP) are calculated by relating the number of genes throughout the genome with a particular function (noted OBS), the number of genes in the region of interest (noted REG) and the total number of genes in the genome (noted TOT):

$$ \mathrm{E}\mathrm{X}\mathrm{P}=\frac{\mathrm{OBS}\times \mathrm{R}\mathrm{E}\mathrm{G}}{\mathrm{TOT}} $$

Then calculating the frequency for each GO term in each region is made and observed numbers are compared to the expected values calculated through chi-square tests for the same GO term between the three compartments.

### Evolutionary origin of genes

To investigate the evolutionary origin of genes in the three genomic compartments of the genome, we used three sets of genes that derive from phylogenetic reconstructions [21]. The first set comprises the genes that were present in the Asterid ancestor (named ANC2), the second set includes the genes existing in the Solanum ancestor (named ANC1), and the third set encompasses genes that are specific to tomato (Heinz 1706) (named NEW). Data were obtained by courtesy of Alexandra Louis (Ecole Normale Supérieure, Paris, France).

Expected values (noted EXP) are calculated by linking the number of genes in the overall genome having a particular evolutionary origin (noted OBS), the number of genes in the region of interest (noted REG) and the total number of genes in the genome (noted TOT):

$$ \mathrm{E}\mathrm{X}\mathrm{P}=\frac{\mathrm{OBS}\times \mathrm{R}\mathrm{E}\mathrm{G}}{\mathrm{TOT}} $$

The distribution of these different groups in the three types of genomic regions was then observed as a histogram and statistical analysis checking the fit between the observed and the expected distribution was made with R through such chi-square tests.

### Distribution of stress genes

We studied the distribution of a list of known stress genes in tomato (from [20]) within the three genomic regions RP, INT and RR. Each gene has then been assigned its belonging region based on its genomic coordinates, and counting has subsequently been achieved. To assess whether the distribution of these genes correspond to the expected distribution in each compartment based on their gene content, calculation of theoretical numbers and statistical analysis of chi-square type were performed.

The expected values (noted EXP) is calculated by linking the number of genes identified as stress response genes (noted OBS), the number of genes in the region of interest (noted REG) and the total number of genes in the genome (noted TOT):

$$ \mathrm{E}\mathrm{X}\mathrm{P}=\frac{\mathrm{OBS}\times \mathrm{R}\mathrm{E}\mathrm{G}}{\mathrm{TOT}} $$

### Detection of repeat methylations

Methylations location data into the genome were available for three different methylation contexts: CG, CHH, CWG. Using a manual script, for each repeat involved in an association, we verified that it was methylated or not and what was the context of this methylation. For this, and for each repeat, each position of it has been sought in the methylation file to obtain the information necessary for the analysis.

### Associations (repeat / gene, DMR / TE and DMR / RIN)

Several types of associations between genes and transposable elements were defined for the analysis of expression: an upstream association (TE witchin 1 kb upstream of the gene), a downstream association (TE within 1 kb downstream of the gene) and an association for genes with a TE overlapping at least one of their introns.

To detect each of these associations, the BEDtools [28] *closest* tool has been used with different options as required: −*io* to ignore overlapping, −*iu* to ignore upstream associations, and *-id* to ignore downstream associations. The associations of interest are selected depending on the distance, between the gene and the nearest repetition, provided by the software in the last column of the result file. In the case of DMR / TE or DMR / RIN associations, only overlaps between the two types of sequences were selected with R after using *bedtools closest*.

To analyze the expression of genes associated with repeats, only genes with a single repeat close to their sequence, i.e. less than 1 kb, have been preserved. Once associations were identified, Mann Witney statistical tests with continuity correction (*wilcox.test()* in R) were performed to see if expression differences are observable according to the location (upstream, downstream, or in a intron) of repeat near gene.

The analysis of enrichment of copies annotated by each consensus of repetitive elements nearby differentially regulated genes was performed according to the methodology used by Makarevitch on maize (see [22]). For each repetitive element present at least once upstream of a gene, we counted the number of s copies observed upstream of genes that are up-regulated, down-regulated and stable. From this table, we calculate the theoretical values of this distribution for each consensus. For this, a calculation involving the number of observations of a consensus for each transcriptional status (up, down, stable) (REGUL_CSS), the total number observed in the genome for the same consensus (EFF_CSS) and the total number for all the consensus for the type of regulation studied is performed (REGUL_TOTAL):

$$ \mathrm{THEO}=\frac{\mathrm{REGUL}\_\mathrm{C}\mathrm{S}\mathrm{S} \times \mathrm{EFF}\_\mathrm{C}\mathrm{S}\mathrm{S}}{\mathrm{REGUL}\_\mathrm{TOTAL}} $$

Statistical analysis checking the fit between the observed and the expected distribution was made with R through such chi-square tests. Finally, a filtering of the results is performed to retain as valid, the consensus associated with at least 10 genes expressed, with an enrichment of the observed value at least twice the expected value and a *p*-value less than 0,001.

For dynamic genome analysis, concerning genes associated with repeat-associated DMRs, all the associations found were preserved, a gene can then be associated with several repeat–associated DMRs. Mann Witney statistical tests with continuity correction (*wilcox.test()* in R) were then carried out to examine differences in gene expression between different classes in pairs.

### Graphs and statistical analyzes

The circular graphs in this article were created with the tool *Circos* [27], all other graphics have meanwhile been established under *R v3.0.2* with *ggplot2* library.

Statistical analyzes were also performed with R v3.0.2 using the commands *chisqtest()*, to test the suitability of a data series for a family of probability laws or testing the independence between two random variables, and *wilcox.test()*, that tests the hypothesis that the distribution of data is the same in both groups defined. A Bonferroni correction was applied to analyze that require multiple comparisons.

### JBrowse

Coordinates for the repeats identified in this paper using REPET, RepeatScout, Tallymer were converted to SL2.50 reference space from SL2.40 using a script (https://github.com/solgenomics/Bio-GenomeUpdate/blob/master/update_coordinates_gff.pl) and chromosome accessioned genome path files for SL2.40 and SL2.50 assemblies (ftp://ftp.solgenomics.net/tomato_genome/wgs/assembly/). Please note that 15 repeats identified by REPET, RepeatScout and Tallymer in SL2.40 were not ported over to SL2.50 since they straddled scaffolds in SL2.40 that were rearranged in SL2.50. All the repeats mapped to SL2.50 are available for analysis in the JBrowse genome browser at https://solgenomics.net/jbrowse.

### RIN binding site analysis

A RIN binding sites analysis was performed using the binding peaks provided in data from Zhong et al. [10], in the Table S8, column “RIN binding peak”, and which have been extended to plus or minus 10 bp. First, we determined which RIN sites are associated with DMRs with the command line *bedtools closest -d -a RINs -b DMRs* from the BEDtools software [28]. RIN binding sites and DMRs involved in overlap were then identified and recovered in R, in bed files, column 19 indicates the distance between the two entities, so we selected distance equal to 0.

For DMRs involved in these associations, we then searched for those associated to repeats, using *bedtools closest -d -a DMRs -b TEs* command line again. The results of interest, i.e. the overlap between DMRS and repeats, were again identified and recovered in R (column 19 of the table is equal to 0).

A second analysis of these RIN binding sites was conducted in the same procedure, but this time, by extending the area of interest around the binding peak at plus or minus 500 bp because DMRs are most often next to RIN binding sites and non-overlapping.

## Abbreviations

d.p.a., days post anthesis; DMRs, differentially methylated regions; EVEs, endogenous viral elements; SSRs, simple sequence repeats; TEs, transposable elements

## Additional files

Additional file 1: Figure S1. Repeats composition of the genome of *S. lycopersicum*. The different families of repeats have been defined according to the Wicker’s classification. The percentage of coverage of each family is calculated relative to the total coverage of the genome by repeats. (TIF 321 kb) Additional file 2
**Table S1.** Genome coverage of the different repeat families. For each repeat family, a coverage calculation has been performed and the percentage of coverage of each family was calculated.by relating the cover of every family with respect to the overall repeat coverage of the genome. **Table S2.** Compartmentalization of the tomato genome in three major regions. Coordinates in gff3 format of the three regions of the genome based on the repeats and genes coverage calculation into 500 kb windows with overlap of 50 kb.**Table S3.** Names of identified TE-genes. List of ID and name of each gene identified as a TE-gene. **Table S4.** Statistical results of the analysis of transposable elements consensus by the method of Makarevitch and al. **Table S5.** Functional annotation of differentially expressed genes candidate as RIN targets. (XLSX 89 kb) Additional file 3: Figure S2. Three categories of genomic regions. K-mean clustering results considering CDS and TE percentage of coverage of each window. We choose to defined three types of regions based on that result. (TIF 61 kb) Additional file 4: Figure S3. Deviation of genome coverage by the three major repeat families. This boxplot shows the deviation of the genome coverage to the mean value of the three main repeat families of the tomato genome, Gypsy, Copia and Class II elements (bringing together the elements DNA and DNAna). The coverage is calculated by window of 500 kb with an overlap of 50 kb and standardized values are determined based on these calculations. Positive values reflect an enrichment while negative values reflect depletion of that type of repeat. Chi-square *P* values < 0.001 for each family versus others except for Copia in RR. (TIF 97 kb) Additional file 5: Figure S4. GC content of repeats. (**A**) For each genomic compartment, the percentage of GC in repeats have been calculated. (**B**) Percentage of GC content in the four main families of repeats in tomato genome. (TIF 116 kb) Additional file 6: Figure S5. TE-genes identification. (**A**) Proportion of putative TE-genes and true genes genes in each genomic region of the genome. (**B**) Comparison of the expression of genes and TE-genes in each genomic region. (TIF 48 kb) Additional file 7: Figure S6. Distance between repeats and genes varies depending on genomic region. After determining for each gene the nearest repeat upstream and downstream to their sequence, we compare these distances between the three genomic regions. (TIF 101 kb) Additional file 8: Figure S7. Gene expression levels depending on DNA methylation. Gene expression depending on the location of the repetition and status methylated or not. The four graphs correspond to the three main methylation contexts (CHH, CG, CHG) and all methylation contexts without distinction (All contexts). Mann Whitney statistical analyzes were conducted to test the differences observed and the results are shown in the the « All contexts » with a different symbol depending on the value of the *P*-value. (TIF 275 kb) Additional file 9: Figure S8. The age of the genes specific to each genomic region. Counting genes considering their phylogenetic origin and comparing that repartition to that expected give us an information about gene age repartition in the three compartments. Statistical analyzes (chi-square tests) were conducted to validate the observations and are represented by the *P*-value on this graphic. (TIF 130 kb) Additional file 10: Figure S9. Distribution of associations between repeats and DMRs in the different repeat families. After determining the associations between repeats and DMRs, counting of each family has been achieved and the percentage was defined by relating this count to the total number of defined associations. (TIF 146 kb)
