# Supplementary figures and images for: Comprehensive repeatome annotation reveals strong potential impact of repetitive elements on tomato ripening
Source: BMC Genomics. 2016 Aug 12;17:624. doi: 10.1186/s12864-016-2980-z (PMC4981986; doi:10.1186/s12864-016-2980-z)

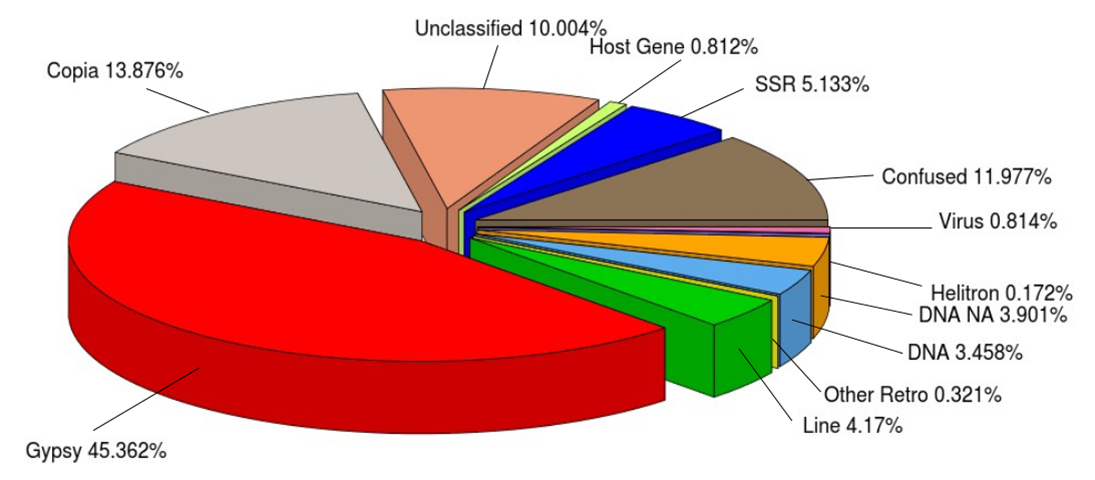

Supplement: Additional file 1: Figure S1. — Repeats composition of the genome of S. lycopersicum. The different families of repeats have been defined according to the Wicker’s classification. The percentage of coverage of each family is calculated relative to the total coverage of the genome by repeats. (TIF 321 kb) [file 12864_2016_2980_MOESM1_ESM.tif]

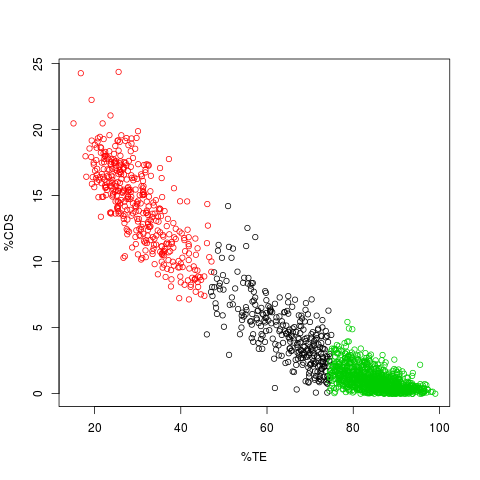

Supplement: Additional file 3: Figure S2. — Three categories of genomic regions. K-mean clustering results considering CDS and TE percentage of coverage of each window. We choose to defined three types of regions based on that result. (TIF 61 kb) [file 12864_2016_2980_MOESM3_ESM.tif]

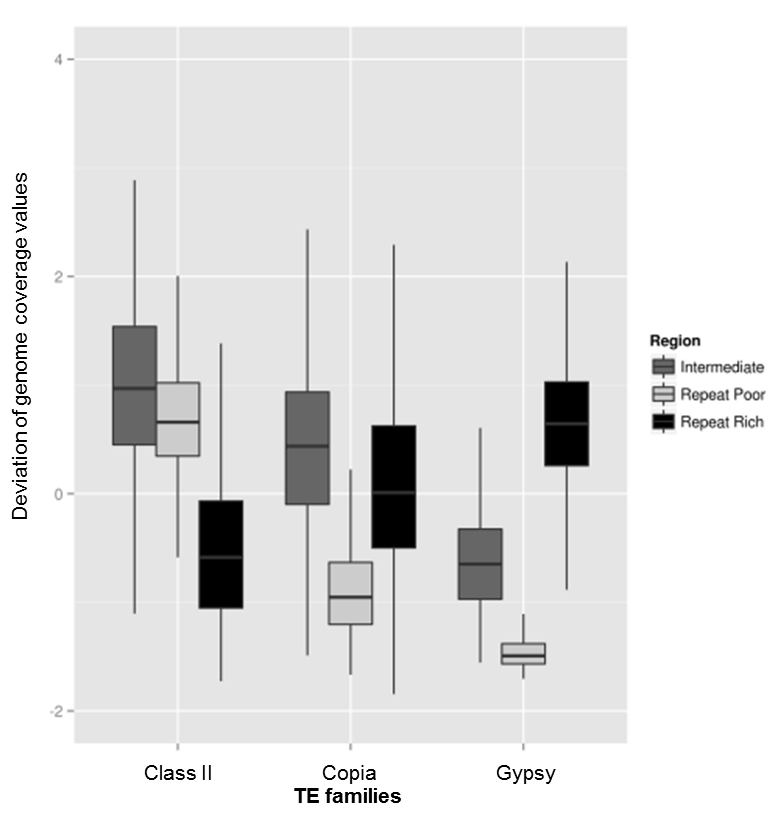

Supplement: Additional file 4: Figure S3. — Deviation of genome coverage by the three major repeat families. This boxplot shows the deviation of the genome coverage to the mean value of the three main repeat families of the tomato genome, Gypsy, Copia and Class II elements (bringing together the elements DNA and DNAna). The coverage is calculated by window of 500 kb with an overlap of 50 kb and standardized values are determined based on these calculations. Positive values reflect an enrichment while negative values reflect depletion of that type of repeat. Chi-square P values < 0.001 for each family versus others except for Copia in RR. (TIF 97 kb) [file 12864_2016_2980_MOESM4_ESM.tif]

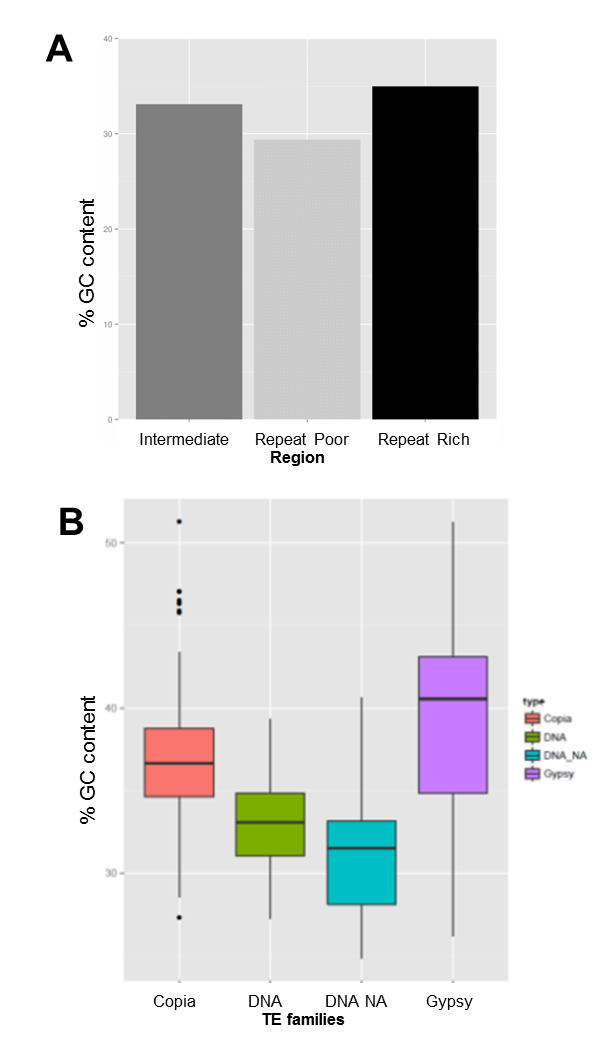

Supplement: Additional file 5: Figure S4. — GC content of repeats. (A) For each genomic compartment, the percentage of GC in repeats have been calculated. (B) Percentage of GC content in the four main families of repeats in tomato genome. (TIF 116 kb) [file 12864_2016_2980_MOESM5_ESM.tif]

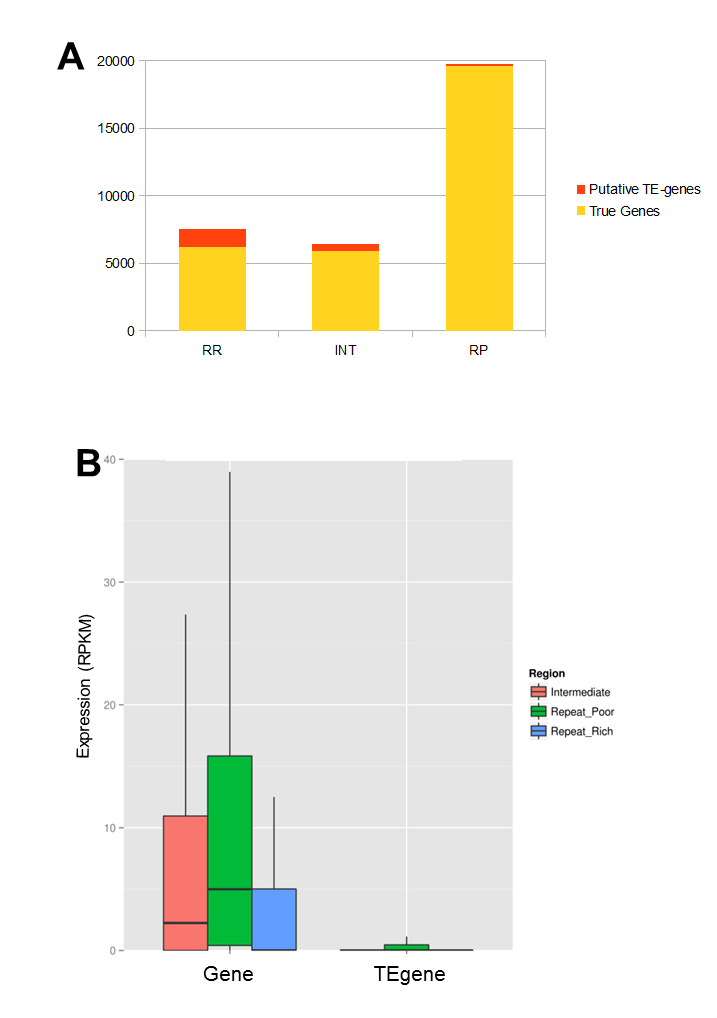

Supplement: Additional file 6: Figure S5. — TE-genes identification. (A) Proportion of putative TE-genes and true genes genes in each genomic region of the genome. (B) Comparison of the expression of genes and TE-genes in each genomic region. (TIF 48 kb) [file 12864_2016_2980_MOESM6_ESM.tif]

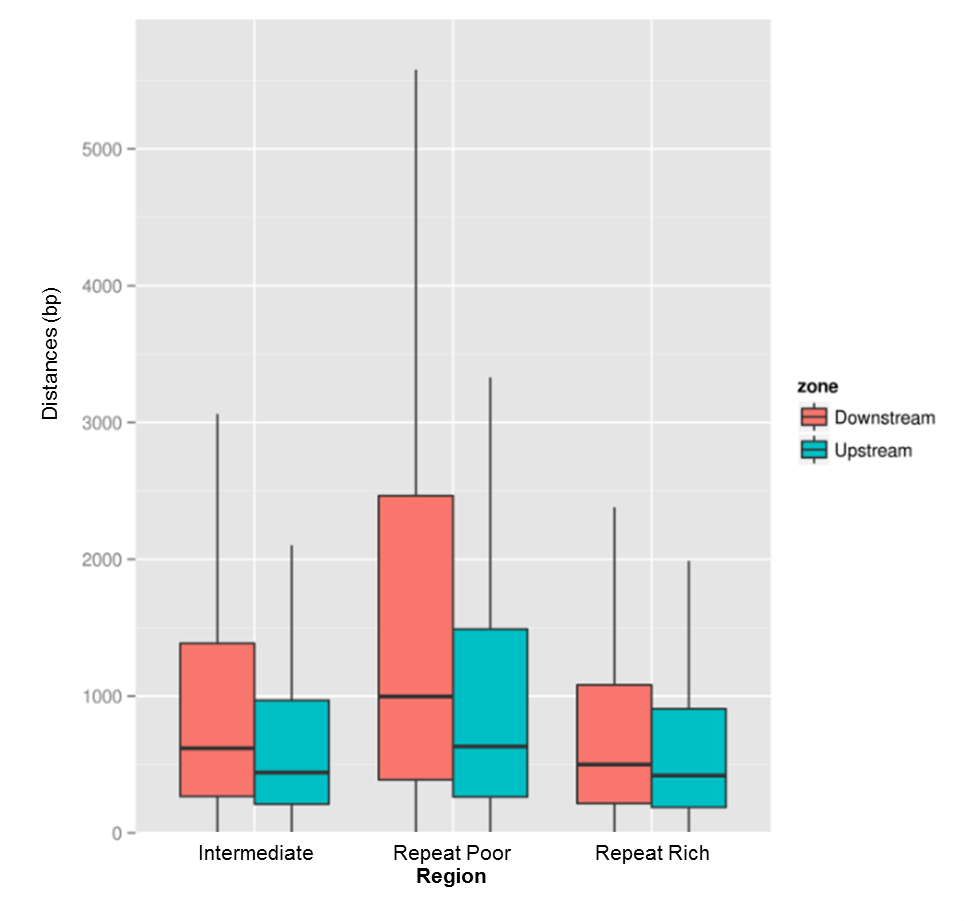

Supplement: Additional file 7: Figure S6. — Distance between repeats and genes varies depending on genomic region. After determining for each gene the nearest repeat upstream and downstream to their sequence, we compare these distances between the three genomic regions. (TIF 101 kb) [file 12864_2016_2980_MOESM7_ESM.tif]

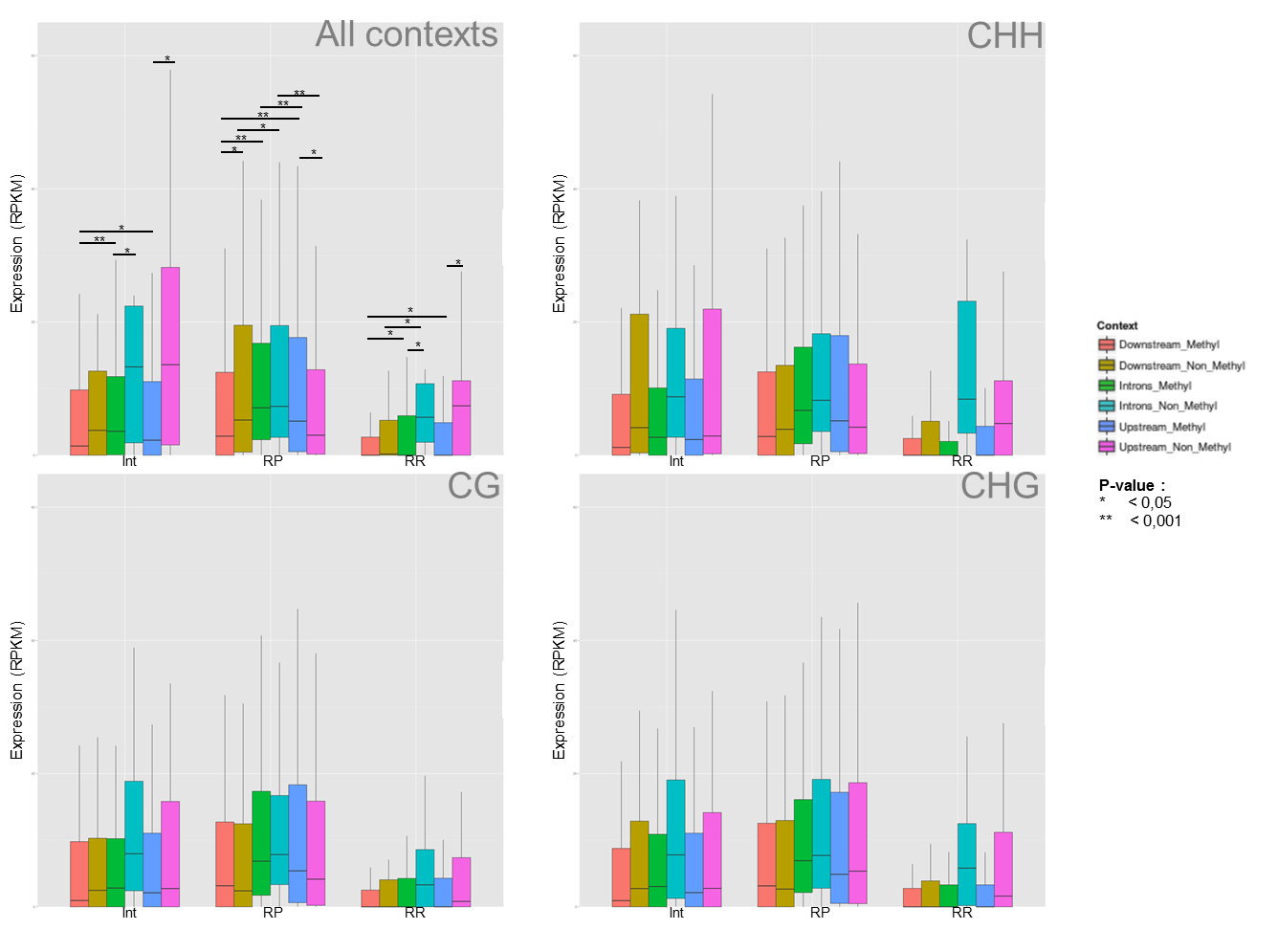

Supplement: Additional file 8: Figure S7. — Gene expression levels depending on DNA methylation. Gene expression depending on the location of the repetition and status methylated or not. The four graphs correspond to the three main methylation contexts (CHH, CG, CHG) and all methylation contexts without distinction (All contexts). Mann Whitney statistical analyzes were conducted to test the differences observed and the results are shown in the the « All contexts » with a different symbol depending on the value of the P-value. (TIF 275 kb) [file 12864_2016_2980_MOESM8_ESM.tif]

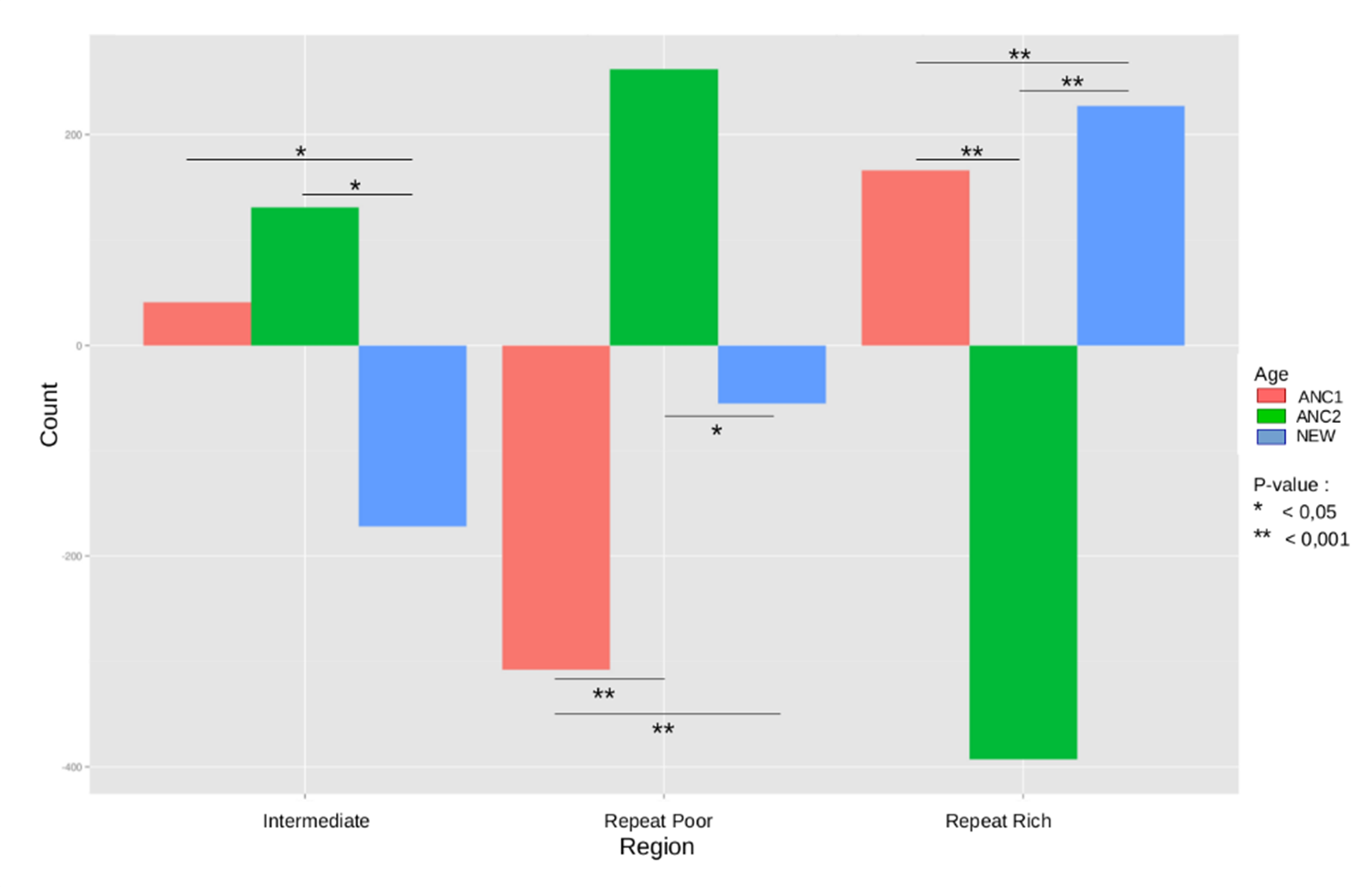

Supplement: Additional file 9: Figure S8. — The age of the genes specific to each genomic region. Counting genes considering their phylogenetic origin and comparing that repartition to that expected give us an information about gene age repartition in the three compartments. Statistical analyzes (chi-square tests) were conducted to validate the observations and are represented by the P-value on this graphic. (TIF 130 kb) [file 12864_2016_2980_MOESM9_ESM.tif]

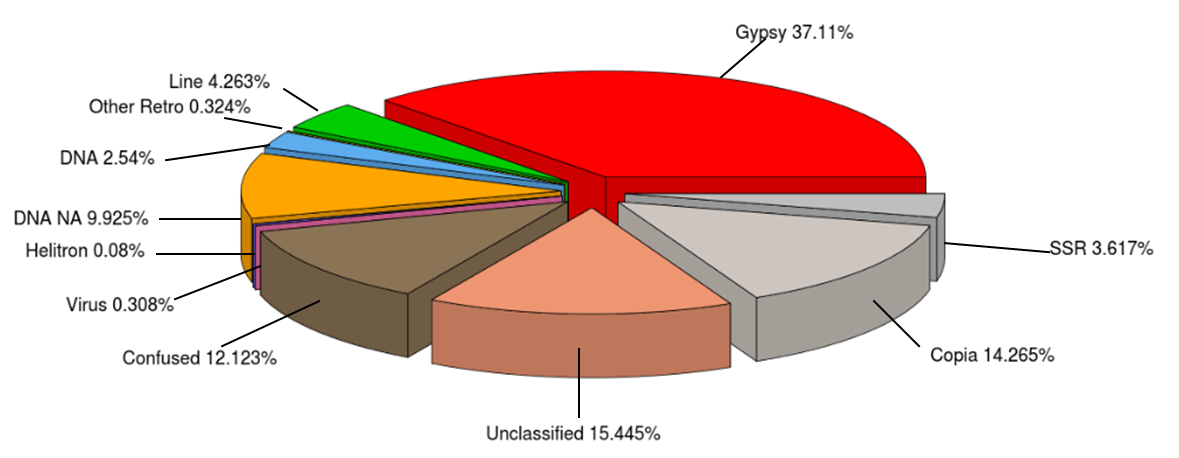

Supplement: Additional file 10: Figure S9. — Distribution of associations between repeats and DMRs in the different repeat families. After determining the associations between repeats and DMRs, counting of each family has been achieved and the percentage was defined by relating this count to the total number of defined associations. (TIF 146 kb) [file 12864_2016_2980_MOESM10_ESM.tif]
